# Dynamics of the HIV Epidemic in MSM

**DOI:** 10.1155/2014/497543

**Published:** 2014-06-29

**Authors:** Yujiang Jia, Muktar H. Aliyu, Z. Jennifer Huang

**Affiliations:** ^1^HIV/AIDS, Hepatitis, STD and TB Administration, The District of Columbia Department of Health, Washington, DC 20002, USA; ^2^Vanderbilt Institute for Global Health and Department of Health Policy, Vanderbilt University, Nashville, TN, USA; ^3^Department of International Health, Georgetown University, Washington, DC 20057, USA

This special issue documents a unique pattern of the HIV epidemic and its associated factors among men who have sex with men (MSM) in China. In high-income countries AIDS has disproportionately impacted MSM. MSM account for the preponderance of prevalent AIDS cases in the United States, Canada, the European Union, Australia, and New Zealand [1–11]. In contrast, in many low- and middle-income countries the HIV epidemic is driven by heterosexual sex, injection drug use, and/or contaminated blood collection and transfusion, with MSM comprising a small proportion of all HIV cases [10–12]. However, recent data show a trend of increase in HIV cases among MSM in Asia, Africa, South America, and Eastern Europe [8–16]. China is one of the countries facing the challenge of an emerging HIV epidemic in MSM. In China, rates of HIV infection in injecting drug users have fallen, remained stable and at low levels in female sex workers, but markedly increased in MSM. The latest national report revealed that the proportion of newly diagnosed HIV cases due to male homosexual contact has increased from 12.2% in 2007 to 32.5% in 2009; while the national HIV prevalence among MSM had a 4.5-fold increase in the past ten years (i.e., from 1.4% in 2001 to 6.3% in 2011) [17]. HIV cases resulting from transmission associated with illegal blood donations have been largely eliminated following the crackdown on unscrupulous blood donors in the late 1990s. The purpose of this special issue is to improve our understanding of the dynamics of the HIV epidemic and its associated factors that are driving the epidemic among MSM in China.

One of the papers in this special issue is by Y. Zhou and colleagues and it describes differences in the prevalence of HIV and syphilis among MSM living in Chinese cities with differing levels of economic development. The authors report a pooled prevalence of 6.5% among MSM nationally, with higher rates in economically less developed cities than in the developed cities. The paper by E. P. F. Chow et al. on the other hand reveals that HIV prevalence among Chinese MSM has increased rapidly in all Chinese regions in recent years. One of the papers summarizes findings from three cross-sectional surveys in Beijing that demonstrate a disturbing rise in HIV incidence among MSM in Beijing. Y. Zeng and colleagues suggest that this epidemiologic trend is not limited to Beijing, as their paper revealed an increase in HIV prevalence from 13.0% to 19.7% from 2006 to 2013 with an increase of 1.0% per year among MSM in Chongqing. These findings support the inference that MSM in China have become an important risk group that should be targeted by HIV prevention programs.

Risk factors associated with HIV among MSM in China are multidimensional. Such factors may be biological (sexually transmitted diseases, e.g., syphilis), behavioral (unprotected sexual behaviors, overlapping bisexual and commercial sex, and increasing substance use), or sociocultural/environmental (e.g., migration, stigma, and social support). Emphasis of familial tie and procreation in Chinese culture put pressure on MSM to lead a double life and conceal their sexual orientation to family. Consequently, MSM in China often encounter stigma and discrimination. Effective intervention and control measures for HIV need to take all of these multidimensional factors into account. In their contribution, E. P. F. Chow and colleagues review the involvement of MSM in high-risk activities (e.g., commercial sex and intravenous drug use) and the community and governmental responses to the HIV epidemic among Chinese MSM. D. Li and colleagues found that recent HIV infection is associated with bisexual activity and a negative attitude towards safe sex.

Unprotected anal intercourse is a recognized risk factor for HIV transmission in MSM. J. T. F. Lau et al. report geographic variations in factors associated with unprotected anal intercourse among MSM in Shenzhen and Hong Kong. In Shenzhen such factors included being able to find someone to share one's sexual orientation, disclosure of sexual orientation to family members, HIV risk perception, and alcohol or drug use, while disclosure of sexual orientation to family members was the only significant factor identified among Hong Kong MSM.

Recreational drug and alcohol use has increased considerably in China in the past three decades, accompanying a rapidly expanding economy, urbanization, and globalization. The global literature suggests that drug and alcohol use is associated with sexual risk behaviors. The independent association of nitrite inhalant use with more casual sex partners and HIV infection reported by D. Li and colleagues underscores the need for interventions targeting nitrite inhalant use. Y. Liu et al. also show that MSM who consumed alcohol more than once per week were more likely to use illicit drugs, have sex with women, have unprotected insertive or receptive anal sex with men, have more than ten lifetime male sex partners, predominantly practice insertive anal sex, and trade sex for money. M. Liao and colleagues had similar findings, with more frequent episodes of alcohol use being independently associated with unprotected anal sex, bisexual identity, multiple male sex partners, drug use, and higher levels of HIV/AIDS-related stigma and discrimination. Taken together, these findings provide the basis for strengthening alcohol use prevention and risk reduction initiatives among MSM as part of a comprehensive HIV risk reduction approach and for further exploring the interaction between alcohol use and HIV transmission.

Chinese traditional culture emphasizes familial responsibilities; homosexual preferences are highly stigmatized and MSM face strong social pressure to conceal their sexual orientation. Stigma surrounding HIV/AIDS is a barrier to HIV prevention, treatment, and care. People who hold stigmatizing attitudes are also less likely to adopt preventive behaviors and more likely to have multiple sexual partners, a commercial sex partner, and engage in other HIV-related high risk behaviors. D. Huang et al. report low levels of stigmatizing attitudes to be associated with uptake of HIV testing services and utilization of free condoms/lubricants. The authors stress the importance of addressing HIV/AIDS-related stigmatizing/discriminatory attitudes and other barriers when delivering HIV-related interventions and testing services.

The Chinese government has significantly scaled up HIV surveillance and prevention efforts among MSM over the past decade, including the use of community-based approaches via grassroots organizations. However, the lack of an enabling legal and financial environment undermines the role of community-based organizations in HIV surveillance and prevention. The hidden nature of homosexual activity coupled with the prevailing stigma and discrimination hinders the successful delivery of timely, high-quality and effective HIV prevention, care and treatment services that are responsive to the unique needs of MSM in China. We hope that the knowledge generated by the articles in this special issue will contribute to the development of innovative HIV prevention programs that will do just that.

*Yujiang Jia* *Muktar H. Aliyu* *Z. Jennifer Huang*
